# Re-classification within the serogroups O3 and O8 of *Citrobacter* strains

**DOI:** 10.1186/s12866-017-1078-3

**Published:** 2017-07-27

**Authors:** Ewa Katzenellenbogen, Magdalena Staniszewska, Nina A. Kocharova, Małgorzata Mieszała, Agnieszka Korzeniowska-Kowal, Sabina Górska, Yuriy A. Knirel, Andrzej Gamian

**Affiliations:** 1 Hirszfeld Institute of Immunology and Experimental Therapy, Polish Academy of Sciences, Weigla 12, 53-114 Wrocław, Poland; 2 Laboratory of Separation and Spectroscopic Method Applications, Centre for Interdisciplinary Research, The John Paul II Catholic University of Lublin, Lublin, Poland; 3 N. D. Zelinsky Institute of Organic Chemistry, Russian Academy of Sciences, Leninsky Prospekt 47, Moscow, 117913 Russian Federation; 4 Department of Medical Biochemistry, Wrocław Medical University, Chałubińskiego 10, 50-368 Wrocław, Poland

**Keywords:** *Citrobacter*, Lipopolysaccharide, O-antigen structure, Serological specificity, Bacterial classification, Enterobacteria

## Abstract

**Background:**

*Citrobacter* strains are opportunistic pathogens often responsible for serious enteric as well as extra-intestinal diseases, and therefore the O-antigenic scheme, still in use in diagnostic identification, should be set for proper serotyping. The structures of more than 30 different *Citrobacter* O-antigens (O-polysaccharide chains of the lipopolysaccharides) of 43 *Citrobacter* O-serogroups have been elucidated so far. However, relationships between strains in several heterogeneous serogroups still need to be clarified by immunochemical studies. These include complex serogroups O3 and O8, represented by 20 and 7 strains, respectively, which are the subject of the present work. Earlier, the O-polysaccharide structures have been determined for *Citrobacter* O3 strain Be35/57 (PCM 1508) and *Citrobacter* O8 strain Be64/57 (PCM 1536).

**Results:**

Serological studies (immunoblotting) carried out on *Citrobacter* lipopolysaccharides from different strains ascribed to serogroups O3 and O8 showed that each of these serogroups should be divided into non-cross-reacting subgroups. Based on the results of chemical analyses and ^1^H and ^13^C NMR spectroscopy the structure of *Citrobacter* O-antigens from strains PCM 1504 (O6) and PCM 1573 (O2) have been established. Chemical data combined with serological analyses showed that several *Citrobacter* strains should be reclassified into other serogroups.

**Conclusions:**

Immunochemical studies carried out on *Citrobacter* LPS, described in this paper, showed the expediency of reclassification of: 1) strains PCM 1504 and PCM 1573 from serogroups O6 and O2 to serogroups O3 and O8, respectively, 2) strains PCM 1503 and PCM 1505 from serogroups O3 and O8 to new serogroups O3a and O8a, respectively.

## Background

Bacteria of the genus *Citrobacter* of the family *Enterobacteriaceae* are normal inhabitants of animal and human intestinal tract. Some *Citrobacter* strains are often associated with extraintestinal disorders, among which the most significant are neonatal meningitis and brain abscess [1, 2].

Serotyping and classification of these bacteria is important for diagnostic purposes. The genus *Citrobacter* is taxonomically most closely related to *Salmonella* and *Escherichia coli.* Currently, strains of the genus *Citrobacter* are classified into 11 genomospecies based on genetic studies [3]. Serological heterogeneity of *Citrobacter* strains is defined by the structure diversity of the O-antigen [4–6], which represents the O-specific polysaccharide chain (OPS) of the cell-surface lipopolysaccharide (LPS). Based on the LPS O-antigens, *Citrobacter* strains are divided into 43 O-serogroups [7] and 20 chemotypes [5].

Structural analysis of the OPS is crucial for unambiguous assignment of serotypes and their cross-reactivity. This is especially important in the case of *Citrobacter* where heterogeneity of strains within particular serogroup is observed and after re-classification some serotypes and O-antigens are overlapping in different species. Structures of the OPS and core domains of LPS are an efficient tool in classification of Gram-negative bacteria. Structural analysis of *Citrobacter* is an example for such approach enabling fine taxonomic classification of these bacteria. Elucidation of structures of over 30 different *Citrobacter* OPS improved the serological classification of strains and explained multiple cross-reactions between *Citrobacter* and other genera of the family *Enterobacteriaceae*, such as *Hafnia*, *Escherichia, Klebsiella*, and *Salmonella* [6, 8] (and refs cited herein). Although many *Citrobacter* OPS structures have been established, in several heterogeneous serogroups the O-antigens require further immunochemical studies.

The present work is devoted to serogroups O3 and O8, represented by 20 and 7 strains, respectively. The OPS structures have been determined for *Citrobacter* O3 strain Be35/57 (PCM 1508) [9] and *Citrobacter* O8 strain Be64/57 (PCM 1536) [10]. Serological studies of number of strains belonging to serogroups O3 and O8 indicated that each of these serogroups can be further divided into two non-cross-reacting subgroups. Moreover, it was shown that two strains from other serogroups, PCM 1504 (O6) and PCM 1573 (O2), should be reclassified into O3 and O8 serogroups, respectively. Two other strains, PCM 1503 and PCM 1505, should be reclassified from serogroups O3 and O8 into new serogroups. These studies prove that the existing O-antigenic scheme used for serotyping, classification and diagnostic purposes needs modifications to make it consistent with the recent taxonomic changes and serological and structural data on the *Citrobacter* O-antigens.

## Methods

### Bacterial strains, cultivation, isolation of LPS and OPS

*Citrobacter* strains listed in Table 1 were derived from the Polish Collection of Microorganisms (PCM) of the L. Hirszfeld Institute of Immunology and Experimental Therapy (Wrocław, Poland). Analysis of bacteria performed by the matrix-assisted laser desorption/ionization time-of-flight mass spectrometry (MALDI-TOF) with standard procedure using Bruker Daltonics UltrafleXtreme spectrometer and Biotyper 3.1 software proved the classification of all strains to *Citrobacter* genus. Bacteria were cultivated in a Davis broth medium supplemented with casein hydrolysate and yeast extract (Difco), with aeration at 37 °C for 24 h, then harvested and freeze-dried.

**Table 1:** Strains used in this work for studies of *Citrobacter* serogroups O3 and O8

Collection number and name			Serotype		
PCM^a^	IHE Be^b^	CDC^c^	PCM^a^	(Lanyi, 1984)^d^	(Miki et al.*,* 1996)^e^
1497	8/50	M.Wright^f^ Na12	3:3,5	3a,3b,1c:5,6	Na12 3:5,6
*1508*	*35/57*	*P.R. Edwards*	*3a,3b,1c:4,5*	*3a,3b,1c:4,5*	*Na2C 3:4,5*
1498	9/50	M.Wright Na11	3:5,6	3a,3b,1c:8,9	Na11 3:8,9
1509	36/57	P.R. Edwards	3a,3b,1c:7,(8),10	3a,3b,1c:7,(8),10	4439 3:7,(8),10
1510	37/57	P.R. Edwards	3a,3b,1c:8	3a,3b,1c:8	Na22 3:8
1511	38/57	P.R. Edwards	3a,3b,1c:8,9	3a,3b,1c:8,9	Na11 3:8,9
1512	39/57	P.R. Edwards	3a,3b,1c:(9),13,14	3a,3b,1c:(9),13,14	4203–54 3:(9),13,14
1499	10/50	M.Wright Mich5	3:12	3a,3b,1c:39	Mich 5 3:39
1513	40/57	P.R. Edwards	3a,3b,1c:14,15,16	3a,3b,1c:14,15,16	1170–50 3:14,15,16
1514	41/57	P.R. Edwards	3a,3b,1c:(13),17	3a,3b,1c:(13),17	Mich 7 3:(13),17
1515	42/57	P.R. Edwards	3a,3b,1c:21,22	3a,3b,1c:21,22	Fels 3:21,22
1516	43/57	P.R. Edwards	3a,3b,1c:21,23	3a,3b,1c:21,23	Va Ankers 3:21,22
1517	44/57	P.R. Edwards	3a,3b,1c:21,24	3a,3b,1c:21,24	4850–50 3:21,24
1518	45/57	P.R. Edwards	3a,3b,1c:(21),25,27	3a,3b,1c:(21),25,27	1109–51 3:(21),25,27
1519	46/57	P.R. Edwards	3a,3b,1c:(9),29,30	3a,3b,1c:(9),29,30	4045–50 3:(9),29,30
1520	47/57	P.R. Edwards	3a,3b,1c:32,33	3a,3b,1c:32,33	3241–50 3:32,33
1521	48/57	P.R. Edwards	3a,3b,1c:39	3a,3b,1c:39	Mich 5 3:39
1522	49/57	P.R. Edwards	3a,3b,1c:47	3a,3b,1c:47	Ga 97 3:47
*1503*	*14/50*	*M.Wrigth Md 2*	*5:6,9*	*3a,3b,1c or 7,3b,1c*	*Md 2 7:(9),13,14*
*1504*	*15/50*	*M.Wright Mich 10*	*6:15*	*8a,8b:(21),25,26*	*Mich 10 8:(21),25,26*
*1505*	*16/50*	*M.Wright Mich 11*	*6:16*	*8a,1c:32,33*	*Mich 11 8:32,33*
1533	61/57	P.R. Edwards	8a,1c:67	8a,1c:67	Va Singleton 8:67
1534	62/57	P.R. Edwards	8a,8b:8,12	8a,8b:8,12	Fla 1608 8:8,12
1535	63/57	P.R. Edwards	8a,8b:(21),25,26	8a,8b:(21),25,26	Mich10 8:(21),25,26?
*1536*	*64/57*	*P.R. Edwards*	*8a,8b:35,37*	*8a,8b:35,37*	*3080–50 8:35,37*
1572	106/59	P.R. Edwards	8a,8c:35,37	8a,8c:35,37	3080–50 8:35,37?
2539	60/57	P.R. Edwards	8a,1c	8a,1c	(Bonn 5937 8:53,55)?
*1573*	*107/59*	*P.R. Edwards*	*2a,1b:21,22*	*2a,1b:21,22 or 8a,1c:21,22*	*Md 10 2:21,22*
1496	5/50	M.Wright Md 10	2:7	2a,1b:21,22	Md 10 2:21,22

^a^Polish Collection of Microorganisms

^b^Institute of Hygiene and Epidemiology in Prague, Czech

^c^Center for Disease Control in Atlanta, USA

^d^acc. to [22]

^e^acc. to [21]

^f^Source strains of antigenic scheme of West and Edwards corresponding to numbers and names in other collectionsc

^g^O-antigens of strains marked in Italic were subjected to structural studies

The LPS were isolated from bacterial mass by the phenol-water procedure [11], recovered from water phase and purified as described [12]. The LPS (called LPS I) from two strains (Be35/57 and Be64/57) were isolated by dialysis of the phenol-water extract without separation of layers and purified with cold aq 50% trichloroacetic acid to precipitate proteins and nucleic acid [6]. The yield of the LPS was 1.5–2.5% of dry bacterial mass.

LPS was heated with 1% acetic acid at 100 °C for 1–2 h and the carbohydrate-containing supernatant was fractionated by gel filtration chromatography on a Sephadex G-50 Fine column (100 × 2.0 cm) in 0.05 M pyridinium acetate buffer pH 5.4 to obtain a high-molecular mass O-polysaccharide.

### Preparation of sera and immunochemical analysis

Rabbit sera against whole cells of *Citrobacter* strains of serotype O3 (PCM 1497 and PCM 1508) and O8 (PCM 1533, 1536, 1572, 2539) were prepared as described earlier [13]. Sera against cells of strains PCM 1503, 1505 and PCM 1531 were from previous studies [8, 14, 15]. The animal studies were conducted in strict accordance with the ethical guidelines established by the National Ethics Committee and approved by the First Local Ethics Commission at the Institute of Immunology and Experimental Therapy, Polish Academy of Sciences (LKE 53/2009).

SDS-PAGE of LPS was performed by the method of Laemmli [16]. The gels were stained with the silver reagent [17] or immunoblotted according to [18]. Briefly, after separation in SDS-PAGE, the LPS was transblotted from the gel onto a PVDF membrane (Immobilon P, Millipore). The air-dried membrane was incubated with anti-*Citrobacter* serum diluted in TBS-T (20 mM Tris-HCl, 50 mM NaCl, 0.05% Tween-20, pH 7) containing 1% BSA, washed with TBS-T and incubated with goat anti-rabbit IgG conjugated with alkaline phosphatase diluted in TBS-T. The immunoblot was visualised with the staining reagent (nitro-blue tetrazolium and 5-bromo-4-chloro-3-indolyl phosphate in 0.05 M Tris/HCl pH 9.5 containing 5 mM MgCl_2_).

Passive hemagglutination assay was performed as described previously [13]. The LPS were at first heated (1 mg/ml PBS, 100 °C, 2 h) or alkali treated (0.25 M NaOH, 56 °C, 1 h) [19]. The sheep erythrocytes (0.2 ml of packed cells) were coated with a suspension of 1 mg LPS/ml PBS at 37 °C for 1 h. The hemagglutination assay was performed with 1% erythrocytes and ten-fold dilutions of serum, all in PBS at 37 °C for 2 h. Results were expressed as the reciprocal titres of the serum dilutions.

### Sugar and methylation analysis

A sample of the OPS was hydrolyzed with 2 M TFA (120 °C, 2 h), monosaccharides were converted conventionally into the alditol acetates and analyzed by GLC-MS on a Hewlett-Packard 5971A instrument equipped with an HP-1 capillary glass column (12 m × 0.2 mm) using a temperature program of 150 °C (3 min) to 270 °C at 8 °C min^−1^. Methylation of the OPS was performed by the method of Gunnarsson [20]. The partially methylated monosaccharides were derived by hydrolysis of the methylated polysaccharide as in sugar analysis or with 10 M HCl (80 °C, 30 min), converted into the alditol acetates and analyzed by GLC-MS as above.

### NMR spectroscopy

Polysaccharide samples were freeze-dried twice from a 99.9% D_2_O solution and dissolved in 99.95% D_2_O. ^1^H- and ^13^C–NMR spectra were recorded at 53 °C on a Bruker DRX-500 spectrometer (Germany) and chemical shifts are reported with internal acetone (δ_H_ 2.225, δ_С_ 31.45) as reference for calibration. The NMR spectra were recorded and data processed using standard Bruker software.

## Results and discussion

### Serological studies

The strains ascribed by several authors [4, 21, 22] to serogroups O3 and O8 are listed in Table 1. The names of species are used according to Miki et al. [21]. As the serological identity of several strains was ambiguous we have characterized them immunochemically. The passive hemagglutination test of *Citrobacter* O8 LPS with rabbit antisera against the whole bacteria revealed high degree of identity of strains PCM 1536 and PCM 1572, whereas the other LPS studied (PCM 2539) was different (Table 2). LPS of strain PCM 1533 showing low cross reactivity with LPS of PCM 1536 and PCM 1572 was considered as an R form, like LPS of strains PCM 1534 and PCM 1535 but their R character was not proved experimentally.

**Table 2:** Titres of rabbit sera against whole bacteria in passive hemagglutination test with *Citrobacter* O8 LPS. Reciprocal titres in parentheses concern alkali treated LPS used for coating sheep erythrocytes

	anti-2539	anti-1533	anti-1536	anti-1572
LPS 2539 (60/57)	2560 (2560)	0 (0)	0 (0)	0 (0)
LPS 1533 (61/57)	160 (0)	320 (40)	40 (0)	160 (20)
LPS 1536 (64/57)	0 (0)	160 (0)	1280 (5120)	2560 (10240)
LPS 1572 (106/59)	0 (80)	0 (20)	320 (7680)	1280 (20480)

**Fig. 1:**
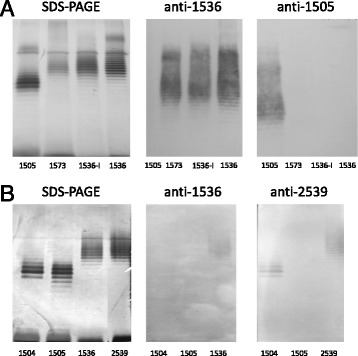
SDS-PAGE and immunoblotting experiments for identification of *Citrobacter* strains in serogroups O8 (panel **a**) and O3 (panel **b**). Panel **a**) Silver-stained SDS-PAGE (*left*) and immunoblotting with anti-*C. braakii*-PCM 1536 (*middle*) and anti-*C. youngae* PCM 1505 (*right*) sera of LPS from *C. youngae* PCM 1505 (lane 1), *C. youngae* PCM 1573 (lane 2), and *C. braakii* PCM 1536 (lane 3 – LPS I [6], lane 4 – LPS). Panel **b**) Silver-stained SDS-PAGE (*left*) and immunoblotting with anti-*C. braakii* PCM 1536 (*middle*) and anti-*C. youngae* PCM 2539 (*right*) sera of LPS from *C. youngae* PCM 1504 (lane 1), *C. youngae* PCM 1505 (lane 2), *C. braakii* PCM 1536 (line 3), and *C. youngae* PCM 2539 (lane 4)

Within the entire serogroup O8 the OPS structure has been determined only for the *Citrobacter* Be64/57 (PCM 1536) [10], thus this strain is considered as a reference for the entire serogroup O8. In the immunoblotting assay (Fig. 1a, middle panel), the anti-1536 serum reacted with the homologous LPS as well as with LPS of strain PCM 1573, which has been originally ascribed to serogroup O2 [23]. This finding shows the expediency of transfer of *Citrobacter* PCM 1573 and PCM 1496 into serogroup O8 (Fig. 1a, Table 1). Data on the structure of the OPS of strain PCM 1573 presented below confirmed this conclusion.

Lipopolysaccharides from strains PCM 1504 and PCM 1505, putative members of serogroup O8 or O6 (Table 1), were not recognized either by anti-1536 (O8) (Fig. 1a and b, middle panels) or anti-1531 (O6) [15] sera. The lack of reactivity of the anti-1505 serum with LPS of strain PCM 1573 (O8) confirms that strain PCM 1505 should not be classified within the serogroup O8 (Fig. 1a, right panel). Moreover, the structural studies revealed the occurrence of D-galactofuranose in the OPS of strain PCM 1505 [14], a component that is not characteristic for serogroup O8. Therefore, *Citrobacter* PCM 1505 should be assigned to a new serogroup.

**Fig. 2:**
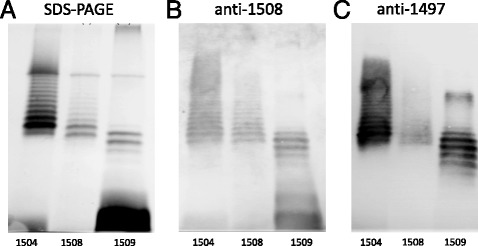
SDS-PAGE and immunoblotting experiments for determination the serotype of *Citrobacter* PCM 1504. Silver-stained SDS-PAGE (**a**) and immunoblotting with anti-*C. youngae* PCM 1508 (**b**) and anti-*C. youngae* PCM 1497 (**c)** sera of LPS from *C. youngae* PCM 1504 (lane 1), *C. youngae* O3 PCM 1508 (lane 2 – LPS I [6]), *C. youngae* O3 PCM 1509 (lane 3)

The other *Citrobacter* strain, namely PCM 2539, has been considered as a member of serogroup O8 (Table 1). The serum anti-2539 reacted with the homologous LPS (Table 2) as well as with LPS of strain PCM 1504 (Fig. 1b, right panel). In turn, the LPS of strain PCM 1504 was recognized by anti-1508 and anti-1497 sera (anti O3 sera; Fig. 2). These data allowed to classify both PCM 2539 and PCM 1504 strains as the members of serogroup O3.

**Fig. 3:**
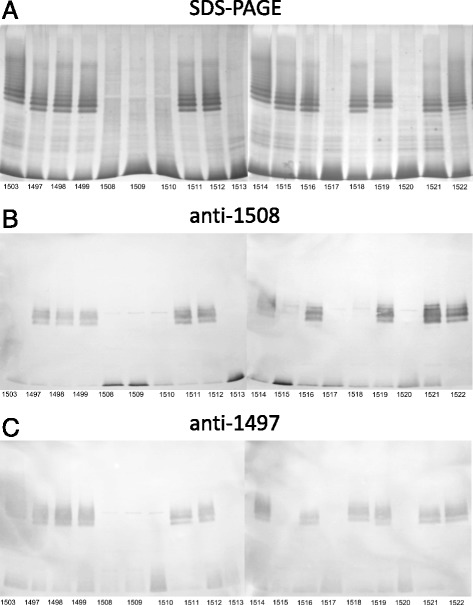
Cross-reactivity of LPS from *Citrobacter* strains classified in O3 serogroup*.* Silver-stained SDS-PAGE (**a**) and immunoblotting with anti-*C. youngae* PCM 1508 (**b**) and anti-*C. youngae* PCM 1497 (**c**) sera of the indicated *Citrobacter* LPS

The serogroup O3 also appeared to be complex. The OPS structure has been determined for *Citrobacter* Be35/57 (PCM 1508) [9] and is considered as a reference structure for the serogroup O3. SDS-PAGE and immunoblotting studies (Fig. 3) showed that the strains of serogroup O3 display diverse pattern and can be divided into several groups. The strains PCM 1497, 1498, 1499, 1511, 1512, 1514, 1516, 1519, 1521, and PCM 1522 were recognized by anti-1508 (O3) serum. Anti-1497 serum reacted additionally with LPS of strains PCM 1503 and PCM 1518 (Fig. 3c). However, anti-1503 serum recognized only LPS of strains PCM 1503 and PCM 1518, indicating that they are serologically different. LPS of the strains PCM 1508, 1509, 1510, 1513, 1517, and PCM 1520 contained low amount of OPS or were already in the R form. In addition, the anti-1503 (not shown) or anti-1508 sera did not recognize the strain PCM 1515 (Fig. 3). The other immunochemical studies on the OPS of *Citrobacter* PCM 1503 [6, 8] and PCM 1505 [14] suggest that these two strains should be reclassified into new serogroups (Table 3). The structural and serological studies presented below indicated that serogroup O3 should be extended by adding strain PCM 1504, which has been ascribed previously to serogroup O6 or O8. In turn, strain PCM 1573 that has been classified previously to serogroup O2, should be transferred to serogroup O8 (Table 1).

**Table 3:**
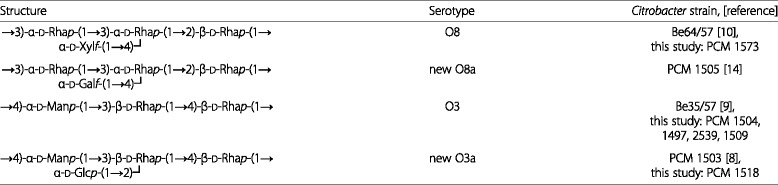
Re-classification of *Citrobacter* strains in serogroups O3 and O8

### Structural analysis of the O-polysaccharides from *Citrobacter* PCM 1573 and PCM 1504 strains

In order to confirm the above serological results, the structural studies were performed on the OPS of strains PCM 1509, 1573, and PCM 1504. The corresponding LPS of *Citrobacter* PCM 1509, 1573 and PCM 1504 strains were recovered in yields 4.6%, 0.4%, and 1.65% of a dry bacterial mass, respectively. In SDS-PAGE, the LPS 1504 and LPS 1573 preparations showed a ladder-like pattern characteristic for LPS of S-type. LPS 1509 preparation in SDS-PAGE experiment has lost its smooth (S) character, therefore it has not been subjected to structural analysis. The mild acid hydrolysis of the LPS followed by fractionation of the carbohydrate material (38%, 59%, and 57% of the LPS weight) on Sephadex G-50 afforded the main fraction P_1_ (OPS), intermediate fraction P_2,_ core oligosaccharide fraction P_3_ and Kdo-containing fraction P_4_. The yields of the OPS and core fractions were 35% and 27.8% for PCM 1504, 53.6% and 22.7% for PCM 1573, and 6.2% and 57.7% for PCM 1509, respectively, of the total material eluted from the column.

**Fig. 4:**
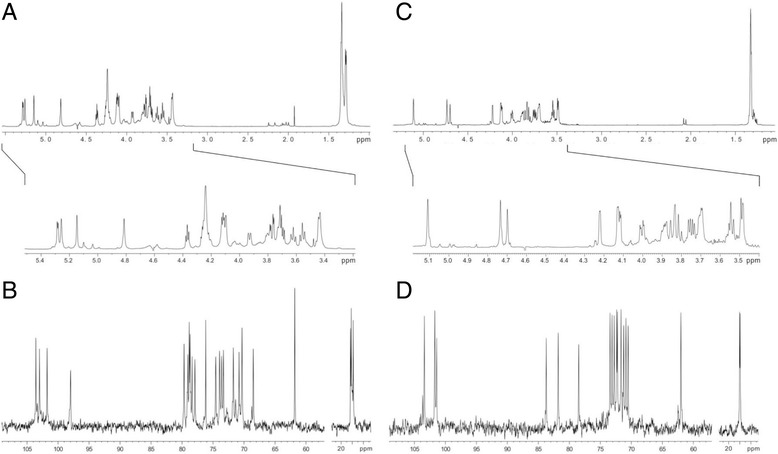
NMR spectra of the isolated OPS. Shown are the ^1^H NMR spectra (**a**, **c**) and ^13^C NMR spectra (**b**, **d**) of the OPS of *Citrobacter* PCM 1573 (**a**, **b**) and PCM 1504 (**c**, **d**). Expanded regions of the ^1^H NMR spectra are shown in the insets

The sugar analysis of the OPS of strain PCM 1573 revealed the presence of two components, namely rhamnose and xylose in molar ratio 3.0:0.8 (hydrolysis with 2 M TFA). Methylation analysis showed the presence of terminal xylofuranose, 2-substituted rhamnopyranose, 3-substituted rhamnopyranose and 2,3-disubstituted rhamnopyranose in molar ratios 0.5:0.9:1.0:1.0 (2 M TFA). The ^1^H NMR and ^13^C NMR spectra of the OPS-1573 (Fig. 4a, b respectively) were identical to those of OPS from strains PCM 1536 [10] and PCM 1572 (data not shown), representing serogroup O8. Therefore, NMR data confirm the results of the chemical analysis and the expediency of classifying strain PCM 1573 into serogroup O8.

Regarding the strain PCM 1504, sugar analysis of the OPS (hydrolysis with 2 M TFA) indicated the presence of rhamnose and mannose in molar ratio 1.9:1.0. Methylation analysis of the OPS revealed the presence of 4-substituted rhamnopyranose, 3-substituted rhamnopyranose, and 4-substituted mannopyranose residues in molar ratios 0.9:0.8:1.0, respectively. Moreover, the ^1^H NMR and ^13^C NMR spectra of the OPS of PCM 1504 strain (Fig. 4c, d, respectively) were identical to the spectra of *Citrobacter* strain Be35/57 [10] belonging to O3 serogroup. Thus, these data exclude the strain PCM 1504 from the O8 serogroup. The performed serological analysis indicated that the original smooth type LPS of strain PCM 1509 was identical to that of strain PCM 1508 (LPS I, O3) (Fig. 2).

## Conclusions

In this paper we report that each of the complex serogroups O3 and O8 can be divided into two non-cross-reacting serogroups. The strains originally ascribed to the serogroups O2 (PCM 1573), O6 (PCM 1504, 1505) and O7 (PCM 1503) that O-antigenic structures are shown in Table 3, should be re-classified into serogroups O3 (PCM 1504) and O8 (PCM 1573). The strains PCM 1503 [8] and PCM 1505 [14] should be classified within new serogroups, namely O3a and O8a, respectively.

## Abbreviations

GLC-MS: Gas-liquid chromatography-mass spectrometry
LPS: Lipopolysaccharide
NMR: Nuclear magnetic resonance
OPS: O-polysaccharide
TFA: Trifluoroacetic acid
