# Erratum to: Re-classification within the serogroups O3 and O8 of Citrobacter strains

**DOI:** 10.1186/s12866-017-1082-7

**Published:** 2017-08-17

**Authors:** Ewa Katzenellenbogen, Magdalena Staniszewska, Nina A. Kocharova, Małgorzata Mieszała, Agnieszka Korzeniowska-Kowal, Sabina Górska, Yuriy A. Knirel, Andrzej Gamian

**Affiliations:** 1 Hirszfeld Institute of Immunology and Experimental Therapy, Polish Academy of Sciences, Wrocław, Poland; 2 Laboratory of Separation and Spectroscopic Method Applications, Centre for Interdisciplinary Research, The John Paul II Catholic University of Lublin, Lublin, Poland; 3 N. D. Zelinsky Institute of Organic Chemistry, Russian Academy of Sciences, Moscow, Russian Federation; 4 Department of Medical Biochemistry, Wrocław Medical University, Wrocław, Poland

## Erratum

Upon publication of the original manuscript [1] it was highlighted that errors had been introduced during the revision process. These errors affect the first column of Table 3. The presentation of the antigenic structures within the first column had been formatted such that they were not clear and were misleading to the reader.

These errors change neither the outcome of the experiments nor the conclusions of the article; and have since been corrected in the original manuscript [1] and acknowledged in this erratum.

The corrected table is shown below:

**Table 3:**
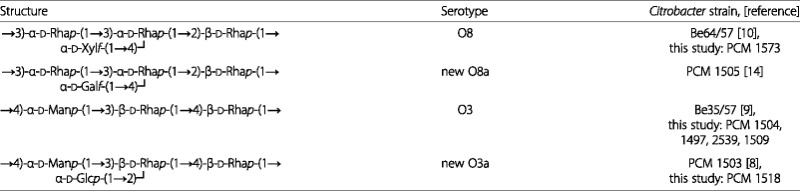
Re-classification of Citrobacter strains in serogroups O3 and O8

The publisher also acknowledges and apologizes for these errors.
